# Impact of COVID-19 and Socioeconomic Status on Delayed Care and Unemployment

**DOI:** 10.1089/heq.2021.0115

**Published:** 2022-02-02

**Authors:** Karina Geranios, Robert Kagabo, Jaewhan Kim

**Affiliations:** ^1^ Dixie State University College of Health Sciences, St. George, Utah, USA.; ^2^ Department of Physical Therapy, University of Utah Health, Salt Lake City, Utah, USA.

**Keywords:** COVID-19, socioeconomic status, delayed care, unemployment

## Abstract

**Background:** Coronavirus disease 2019 (COVID-19) may disproportionately impact minorities and individuals of low socioeconomic status (SES). This study examined unemployment and delayed care due to COVID-19.

**Methods:** Using the Current Population Survey (CPS) from May through October 2020, two questions, namely unable to work and delayed care due to COVID-19, were examined. Unweighted summary statistics and logistic regression were used to analyze the data.

**Results:** A total of 367,950 adult participants 18–64 years old at survey were included. Mean (standard deviation) age was 41 (14) years old, and 36% of the participants had family income ≤$25,000. About 12% reported unable to work and 3% had delayed care. Racial minorities had statistically significant higher likelihood of being unable to work. Individuals with the lowest income, ≤$25,000, had the most serious impact from COVID-19 (odds ratio=1.92, *p*<0.01).

**Conclusion:** Individuals of racial minority groups and lower SES experienced the worst economic outcomes of employment losses.

## Introduction

The coronavirus disease 2019 (COVID-19) pandemic has revealed and exacerbated the inequalities among racial and ethnic minorities in the United States.^1,2^ More than 30 million jobs were lost in the early months of the pandemic,^3^ and both preventative and chronic care management were put on hold, or delayed for many.^4^ Individuals of marginalized backgrounds such as low-income earners were disproportionally affected and resulted in increased health disparities among them.^5^

Racial and ethnic minority populations have generally experienced greater disparities in health care access^6^ and employment in the United States even before the pandemic.^7,8^ These disparities have been pronounced during the COVID-19 pandemic where populations especially those of low socioeconomic status (SES) have experienced heightened effects of delayed health care access and unemployment.^9^ COVID-19 disease affects all populations, however, some demographics such as older persons with preexisting medical conditions and those of certain racial and ethnic minority groups have greater risks compared to the general population.^10–12^ For example, given their percentage make up of the total population, blacks are affected by Covid-19 hospitalizations 1.8 times more than expected.^10–12^

Racial and ethnic minorities are also reported to experience worse outcomes than the general population with blacks and Hispanics twice as likely to die compared to whites.^8,13^ The long-standing disparities of chronic disease, access to health care, systemic racism, housing insecurity, essential jobs on the frontlines, and reliance on public transportation are some of the social inequities also associated with the disproportionate impact of COVID-19 among these populations.^14^

Research on rural communities shows that compared to urban communities, rural communities are less racially and ethnically diverse^15^ and experience worse health outcomes^15,16^ in addition to having less health care access.^15,17^ While there is little research, there is evidence to suggest that rural places too have racial and ethnic health disparities.^15,18^ The COVID-19 pandemic has revealed the severity of these disparities, as ∼33% of rural communities are vulnerable to the impacts of COVID-19 primarily attributed to large elderly populations, higher rates of disability, and significant proportion of uninsured individuals.^19^ For these same reasons, rural areas could experience delayed care attributed to COVID-19.

Given that low-income earners and individuals without a college degree are less likely to telework, these groups have been impacted more in terms of job loss compared to higher income earners and those with college degrees.^20^ The low-income earners have had trouble paying their bills and in some cases used up their financial savings.^20^ The desperate financial impacts could be predicted by income level, and even a year following the start of the pandemic, reports in March 2021 revealed that lower income adults were still experiencing financial hardships and with struggles to rebuild up.^20,21^ However, there is a paucity of evidence on how those with different SES had different impacts from COVID-19.

The hypothesis for this study was that individuals of low SES were more likely to experience severe impacts than those of higher SES. An understanding of disparities of delayed care and unemployment associated with COVID-19 is crucial for policy makers to adopt policies that aim to close these gaps. Using the Current Population Survey (CPS) from May 2020 through October 2020, this study examined the impact of COVID-19 on delayed health care and unemployment among populations across different SES and racial groups.

## Methods

### Data

We used data from the CPS for May-October 2020. CPS is a national monthly house-hold longitudinal survey following participants for 4 months.^4^ The survey's primary goal is to measure unemployment rates and is also widely used for health policy purposes.^22^ Variables in CPS survey include but not limited to age, sex, race/ethnicity, income, marital status, employment/unemployment status, and health insurance coverage. To measure the impact of Covid-19, the May–October 2020 CPS included five supplemental Covid-19-related questions.^4^ Of the five questions, we used two to measure the impact of Covid-19. (1) At any time in the last 4 weeks, were you unable to work because your employer closed or lost business due to the coronavirus pandemic? (2) At any time in the last 4 weeks, did you or anyone in your household need medical care for something other than coronavirus, but not get it because of the coronavirus pandemic?

### Subjects

Participants were adults 18–64 years old at survey time. These participants were included because they were of working age-group and were asked both questions. Participants with no responses or who had missing values in the covariates or the outcome variables were excluded.

### Covariates

Characteristics of the participants were created from the 2020 monthly household core data and the monthly core data. For the monthly core data, age, sex (male and female), race/ethnicity (non-Hispanic white, non-Hispanic black, non-Hispanic others, and Hispanic), marital status (married, divorced/widowed, single), education (high school graduate-Yes or No), any physical/mental difficulty (Yes or No), and family income (≤$25,000, $25,000–$49,999, $50,000–$74,999, $75,000–$99,999, and ≥$100,000) were collected. These covariates were selected especially because prior studies of unemployment and delayed care have used several variables, including, age, sex, race/ethnicity, education, and marital status.^23–25^ The non-metropolitan area (Yes or No) and geographic region (New England, middle Atlantic, east/west north central, south Atlantic, east/west central, mountain, and pacific division) were created using the geographic variables in the monthly household core data. We included the region and division, and the month dummy variables as fixed effects because the outcomes could be different across regions and time.

### Statistical approach

Unweighted summary statistics such as mean, standard deviation (SD), and percent were used to summarize the data. Considering the binary outcomes (unable to work-Yes or No; delayed care-Yes or No), logistic regression was used to estimate odds ratios (ORs) of the covariates with robust standard errors.

Two approaches were used to check the multicollinearity issue. We ran a linear regression with the control variables only (one variable as a dependent variable) and tested a variance inflation factor (VIF). The VIF for the regression models was 1.57, so we did not find any multicollinearity issue. To confirm our results, we used the “collin” Stata command following the logistic regression. From this Stata command, VIF was 1.05. We, however, did not control the categorical variables because the “collin” command allows continuous variables only.

## Results

A total of subjects/participants (unweighted *n*=373,601) was selected from May through October 2020. Of that total, missing in the metropolitan variable (unweighted *n*=3611) and subjects who were not in universe in the delayed care and unable to work questionnaires (unweighted *n*=2040) were excluded. The final sample included unweighted 367,950 subjects. Mean (SD) age of the overall subjects was 41 (14) years, and 49% were male. About 67% were non-Hispanic white and 14.5% were Hispanic. About 36% of the subjects had family income ≤$25,000, and 14.6% family income between $25,000 and $49,999. Among the total subjects, 12.2% reported unable to work due to COVID and 2.93% reported that they had delayed care due to COVID-19 (Table 1).

**Table 1.** Characteristics of Subjects by Unable to Work Status and Delayed Care Status (Unweighted *n*=367,950)

	Unable to work		Delayed care		Total
	No	Yes	No	Yes	
*N*	323,155 (87.8%)	44,795 (12.2%)	357,167 (97.07%)	10,783 (2.93%)	
	Mean (SD)/%	Mean (SD)/%	Mean (SD)/%	Mean (SD)/%	
Age	41(14.3)	40.8 (13.5)	40.8 (14.2)	45.1 (13.7)	41 (14.2)
Male (%)	48.8	48.4	48.9	42.6	48.7
Nonmetropolitan area (%)	18.6	14.8	18.1	19.2	18.2
Race/Ethnicity (%)					
Non-Hispanic white	67.6	59.2	66.5	70.3	66.6
Non-Hispanic black	9.8	11.7	10.1	10	10.1
Non-Hispanic Asian/Pacific Island	7.2	8.6	7.4	5.8	7.4
Non-Hispanic multirace	1.5	1.9	1.5	1.9	1.5
Hispanic	13.9	18.7	14.5	12	14.5
High school graduate (%)	88.5	89.7	88.6	89.9	88.6
Any physical/mental difficulty (%)	8	5	7.2	22.3	7.6
Marital status (%)					
Married	52	47.8	51.5	49.7	51.5
Separated/Divorced/Widowed	13	14.5	13	20.4	13.2
Single	35	37.6	35.5	29.9	35.4
Family income (%)					
≤$25,000	36.7	28.9	35.8	31.9	35.7
$25,000–$49,999	14.7	13.8	14.6	13.4	14.6
$50,000–$74,999	18.7	20.1	18.9	17.4	18.8
$75,000–$99,999	18.5	22.8	19	19.6	19
≥$100,000	11.5	14.4	11.7	17.6	11.9
Region and division (%)					
New England Division	7.8	7.9	7.7	10.8	7.8
Middle Atlantic Division	8.5	10.6	8.7	8.9	8.7
East North Central Division	11	10.6	11	10.2	11
West North Central Division	10.1	7.4	9.9	7.6	9.8
South Atlantic Division	17.7	16.6	17.6	16	17.6
East South Central Division	7.1	5.9	7	6.2	7
West South Central Division	10.5	9.5	10.4	8.9	10.4
Mountain Division	12.6	12.1	12.6	12.4	12.6
Pacific Division	14.6	19.4	15.1	19	15.2
Month (%)					
May	14.3	27.7	15.5	30.9	16
June	14.4	21.2	15	20.4	15.2
July	15.4	16.2	15.5	16.3	15.5
August	16.7	13.2	16.4	12.7	16.3
September	19.2	12	18.5	11.1	18.3
October	20	9.8	19.1	8.6	18.8

SD, standard deviation.

Compared to non-Hispanic white, non-Hispanic black (OR=1.27, *p*<0.01), non-Hispanic Asian/Pacific Islander (OR=1.14, *p*<0.01), and Hispanic (OR=1.40, *p*<0.01) had statistically significantly higher likelihood of being unable to work due to COVID-19 by 27%, 14%, and 40%, respectively (Table 2). Those who were separated/divorced/widowed (OR=1.11, *p*<0.01) and single (OR=1.11, *p*<0.01) had higher odds of being unable to work than those who were married. All groups with less than $100,000 had higher risk in being unable to work than the highest income group (≥$100,000). Specifically, those with the lowest income (≤$25,000) had the most serious impact from COVID-19 compared to any other income group, OR=1.92 (Table 2).

**Table 2.** Factors That Were Associated with Unable to Work

Variable	OR	*p*	95% CI
Age	1.002	0.003	1.001–1.003
Male	1.009	0.577	0.978–1.040
Nonmetropolitan area	0.766	<0.001	0.731–0.802
Race/Ethnicity			
Non-Hispanic white	Reference		
Non-Hispanic black	1.273	<0.001	1.210–1.338
Non-Hispanic Asian/Pacific Island	1.137	<0.001	1.071–1.207
Non-Hispanic multirace	1.404	<0.001	1.242–1.587
Hispanic	1.403	<0.001	1.345–1.463
High school graduate	1.361	<0.001	1.294–1.431
Any physical/mental difficulty	0.528	<0.001	0.491–0.569
Marital status			
Married	Reference		
Separated/Divorced/Widowed	1.111	<0.001	1.059–1.165
Single	1.113	<0.001	1.068–1.159
Family income			
≤$25,000	1.919	<0.001	1.820–2.024
$25,000–$49,999	1.248	<0.001	1.188–1.310
$50,000–$74,999	1.470	<0.001	1.405–1.538
$75,000–$99,999	1.774	<0.001	1.697–1.856
≥$100,000	Reference		
Region and division			
New England Division	Reference		
Middle Atlantic Division	1.075	0.049	1.000–1.155
East North Central Division	0.875	<0.001	0.816–0.939
West North Central Division	0.732	<0.001	0.675–0.793
South Atlantic Division	0.818	<0.001	0.764–0.875
East South Central Division	0.763	<0.001	0.703–0.828
West South Central Division	0.733	<0.001	0.681–0.789
Mountain Division	0.909	0.013	0.844–0.980
Pacific Division	1.081	0.025	1.010–1.157
Month			
May	Reference		
June	0.765	<0.001	0.743–0.787
July	0.556	<0.001	0.538–0.575
August	0.405	<0.001	0.390–0.421
September	0.314	<0.001	0.302–0.327
October	0.236	<0.001	0.226–0.246

CI, confidence interval; OR, odds ratio.

In the delayed care due to COVID-19, all minorities except the non-Hispanic multirace group were less likely to have delayed care than the non-Hispanic white group. The non-Hispanic Asian/Pacific Islander group (OR=0.80, *p*<0.01) and the Hispanic group (OR=0.82, *p*<0.01) had about 20% and 18% less likelihood in the delayed care than the non-Hispanic white group, respectively. The separated/divorced/widowed group had higher risk in the delayed care than the married group by 23% (OR=1.23, *p*<0.01). The middle-income group ($75,000–$99,999) was more likely to have the delayed care than the highest income group (≥$100,000) (OR=1.32, *p*<0.01), but the other income groups did not show statistically significant delayed care than the highest income group (Table 3).

**Table 3.** Factors That Were Associated with Delayed Care

Variable	OR	*p*	95% CI
Age	1.015	<0.001	1.013–1.018
Male	0.782	<0.001	0.742–0.825
Nonmetropolitan area	0.933	0.064	0.866–1.004
Race/Ethnicity			
Non-Hispanic white	Reference		
Non-Hispanic black	0.966	0.453	0.882–1.058
Non-Hispanic Asian/Pacific Island	0.804	<0.001	0.716–0.903
Non-Hispanic multirace	1.196	0.068	0.987–1.448
Hispanic	0.815	<0.001	0.749–0.886
High school graduate	1.083	0.079	0.991–1.183
Any physical/mental difficulty	3.267	<0.001	3.038–3.514
Marital status			
Married	Reference		
Separated/Divorced/Widowed	1.225	<0.001	1.139–1.317
Single	1.015	0.692	0.943–1.092
Family income			
≤$25,000	1.002	0.966	0.920–1.091
$25,000–$49,999	0.996	0.921	0.920–1.078
$50,000–$74,999	1.041	0.309	0.963–1.126
$75,000–$99,999	1.316	<0.001	1.206–1.435
≥$100,000	Reference		
Region and division			
New England Division	Reference		
Middle Atlantic Division	0.792	<0.001	0.707–0.886
East North Central Division	0.669	<0.001	0.600–0.746
West North Central Division	0.617	<0.001	0.544–0.701
South Atlantic Division	0.628	<0.001	0.564–0.699
East South Central Division	0.591	<0.001	0.520–0.672
West South Central Division	0.659	<0.001	0.585–0.742
Mountain Division	0.732	<0.001	0.649–0.824
Pacific Division	0.970	0.566	0.874–1.076
Month			
May	Reference		
June	0.680	<0.001	0.640–0.723
July	0.542	<0.001	0.507–0.580
August	0.407	<0.001	0.378–0.439
September	0.299	<0.001	0.276–0.323
October	0.232	<0.001	0.213–0.252

## Discussion

CPS data pertaining to unemployment and delayed care among racial and ethnic minorities and different income levels show that the health disparities predating the pandemic have widened.^26^ A critical examination of these data is therefore needed to help identify approaches of comprehensive care improvement and lessen the impacts of the disparities. Unemployment in the United States sharply rose with increase of business lockdowns to a record of 14.7% in April 2020, and even with economic standing improvement, unemployment remained as high as 6.9% by October 2020.^27^ Many factors may have contributed to a stark decline in employment, and many of these might be largely underreflected in official unemployment rates. Examples of these factors include lockdowns, closing nonessential businesses, parents staying home with their children to assist with remote learning, voluntary exiting of the workforce due to concerns of contracting Covid-19, and the need to provide caregiving duties.^28^

Our analyses showed that those most impacted by layoffs during the Covid-19 pandemic are characterized as low-income, with earnings less or equal to $25,000 annually. This impact may widen gaps in income inequality. Similar findings have been reported in other studies such as the February 2021, ecological cohort study which examined the relationship between the county-level Gini coefficient, a measure of income inequality in an area, and Covid-19 cases and deaths in the United States.^29^ Counties with more pronounced income inequality had higher numbers of COVID-19 cases and deaths. Confounding factors, such as urban or rural locations, and socioeconomic factors, such as poverty, housing, education level, and health care infrastructure, were accounted for.

The association between Gini coefficient level and COVID-19 impacts were strongest during the summer months, suggesting that low SES individuals, who are more unlikely to work from home and remain working in restaurants, hotels, or entertainment venues, are particularly susceptible to contracting and dying from COVID-19. Targeted interventions, such as expanded public health information campaigns, distribution of personal protective equipment, increased COVID-19 testing, and equitable distribution of the vaccine must be implemented to decrease the risk to those most vulnerable to COVID-19 exposure and complications.

A study published in January 2021 evaluated the relationship between Social Vulnerability Index (SVI), which was developed by the Centers for Disease Control and Prevention (CDC) for counties in the United States and COVID-19 outcomes and found that sociodemographic factors were significantly associated with higher COVID-19 incidence and mortality.^30^ The subindices of populations living in crowded housing, in a single-parent household, or with limited English proficiency, were most impacted, suggesting that low SES populations demonstrate a marked risk of COVID-19 disease impacts.^30^

The Pew's American Trends Panel (ATP) surveyed a nationally representative sample of adults in United States and found that lower income earners were more financially impacted by the COVID-19 recession.^20^ Survey results showed that 46% of lower income Americans had trouble paying their bills since the start of the pandemic, compared to 19% of middle income, and 5% of upper income Americans.^20^ Results also showed that 44% of lower income adults had dipped into retirement savings, 35% had borrowed money from friends or family, 35% received assistance from a charitable organization, and 37% collected government food assistance.^20^ The proportion of those affected in each of these areas appeared to decrease as income level increased. Survey results also showed that 51% of lower income adults reported being able to save less than before the pandemic compared to 35% of middle income, and 21% of upper income adults.^20^

In March 2021, 1 year since COVID-19 was declared a pandemic, people younger than the age of 30 years, without a college degree, female, or black and Hispanic were more likely to experience adverse economic effects than other groups.^21^ Our findings highlight the disproportionate level of economic difficulty experienced by lower income earners due to the COVID-19 pandemic and call for policy ideas aimed at assisting this population to lower the disparity impacts.

In this study results, we found that compared to non-Hispanic whites, minorities experienced disproportionately higher employment impacts due to COVID-19. Hispanics had the worst experience with 40% reporting an inability to work directly due to onset of the COVID-19 pandemic. In addition, non-Hispanic blacks, and non-Hispanic Asians/Pacific Islanders were highly impacted at rates of 27 and 14%, respectively (Table 2). This study's findings are similar to others, for example, one study found that between April and June 2020, risk factors such as having less than a high school education, being a high school graduate, and working in the leisure and hospitality, wholesale and retail trade, or construction industries were strongly associated with COVID-19-induced layoffs.^22^ These sectors employ high percentages of Hispanic, black, and Asian/Pacific Islander groups compared to the majority whites.^31^

The results of this study show the level of delayed care that minority groups experienced was not statistically significantly different compared to the majority non-Hispanic white population. This finding may be explained by knowledge that minority groups generally lack health insurance, are Medicaid beneficiaries, and live in areas with poor health care infrastructure, all of which generally make access to care difficult.^32^ Minority groups may also be young with less comorbidities, or of limited English proficiency resulting in decreased health care services utilization,^33^ while the majority white population are more likely to have adequate health insurance coverage, and live in areas with optimal health care infrastructure, which allows them to participate in preventative and chronic care services.

This is consistent with the National Institute of Health's (NIH) analyses, which confirm these racial and ethnic disparities among utilization of health care services.^33^ Before the COVID-19 pandemic, minorities, such as blacks and Hispanics, were more likely to be uninsured and utilize more emergency room services, but less likely to receive continued preventative care and ambulatory care.^33^

This study also found that delayed care was not statistically different for low-income and high-income earners. These results may also be explained by knowledge that individuals of low SES group backgrounds are generally younger and thus healthier, and less likely to seek or report delaying their access to health care. Data collected before the COVID-19 pandemic indicate that when compared to those using Marketplace insurance, Medicaid beneficiaries, who typically comprise the lowest earners, had fewer clinic office visits, and prescription fillings but more emergency room visits.^33^ The CDC's National Health Interview Survey (NHIS) found that, as of February 2021, uninsured individuals are more likely to have low incomes, live in states without Medicaid Expansion, are young adults, or be black or Latino.^34^ Uninsured individuals also generally have poorer health outcomes and delay primary preventative care, and only seek acute care when they absolutely need it.

There are strengths and limitations associated with this study. CPS data collection and its longitudinal design have been ongoing for many years implying that their survey instruments are valid. The sample size, *n*=367,950, is also large and sufficiently representative of the population, which lends validity to near accurate results and inferences. While the mentioned strengths are good, the May to October period was the first time CPS introduced the COVID-19-related questions and released related data. There is a possibility that vital information that could help explain the results may not have been included in the newly asked questions. While looking into causes of unemployment, there are factors such as lack of childcare that may not be reflected in official numbers and such factors are difficult to account for. Health insurance status could be associated with delayed care. This study, however, did not control for insurance status because this status is not available from the CPS monthly data.

## Conclusion

In the United States, the effects of the economic fallout resulting from the COVID-19 pandemic disproportionately impacted historically disadvantaged populations, especially black, Hispanic/Latino, and those of low SES groups. Using the May to October 2020 CPS data, this study found the loss of employment and income was very severe among ethnic minority and low-income groups. The findings point to a need for prudent policies that may address disparities made worse by the pandemic. Interventions should be informed by an understanding of social safety nets, especially those addressing health care access and economic needs.

## Abbreviations Used

ATP: American Trends Panel
CDC: Centers for Disease Control and Prevention
CI: confidence interval
COVID-19: coronavirus disease 2019
CPS: Current Population Survey
NIH: National Institute of Health's
NHIS: National Health Interview Survey
ORs: odds ratios
SD: standard deviation
SES: socioeconomic status
SVI: Social Vulnerability Index
VIF: variance inflation factor
